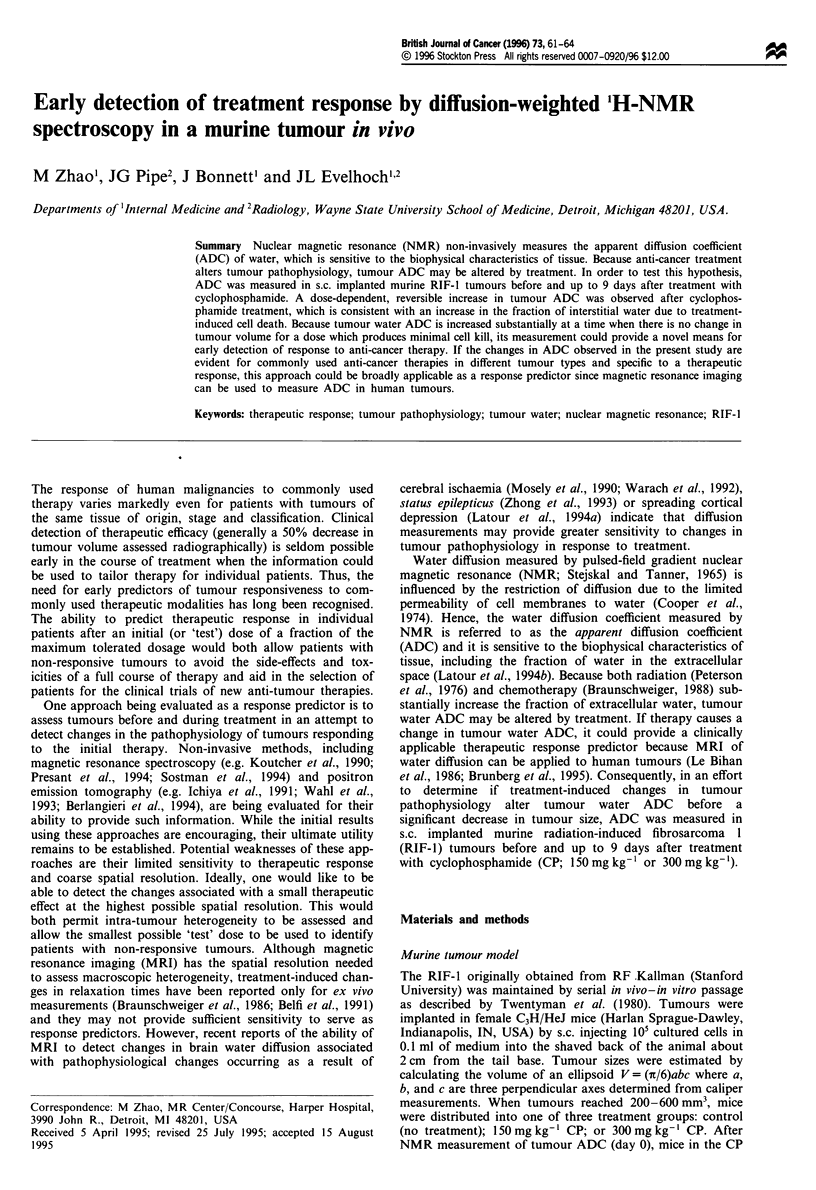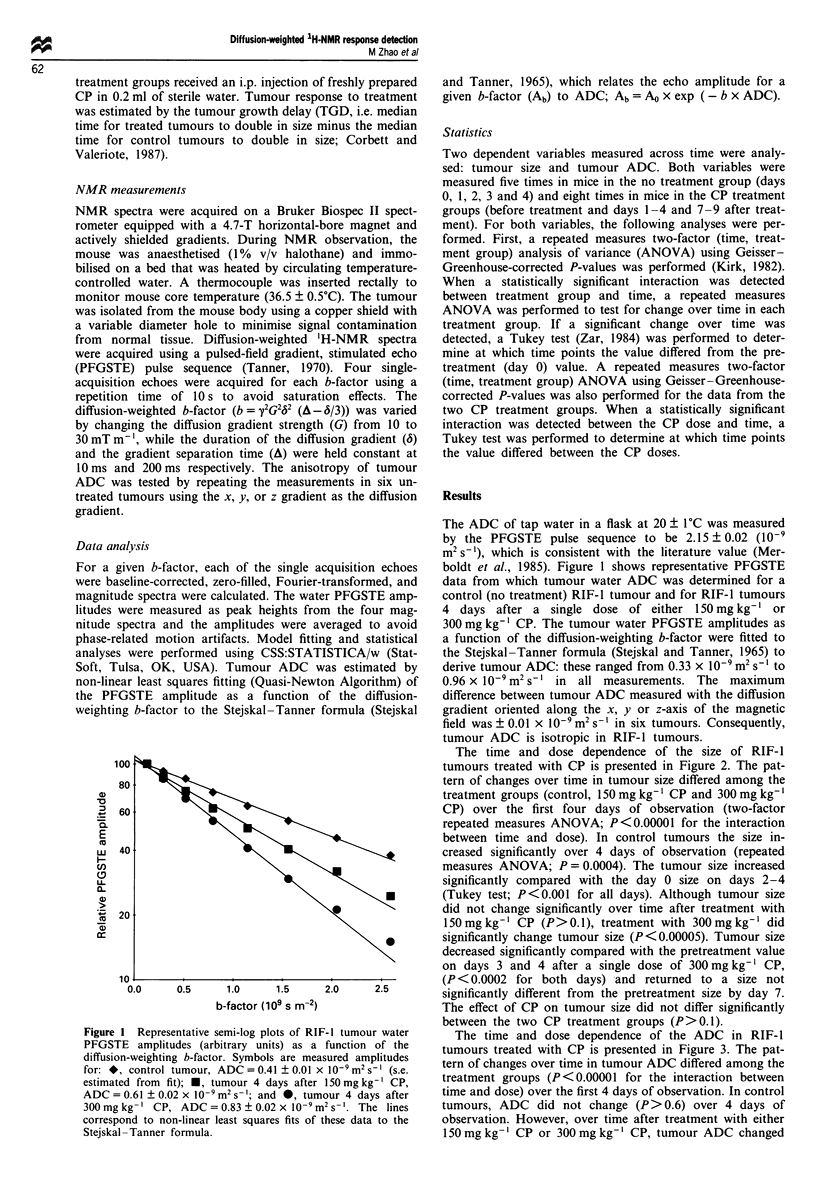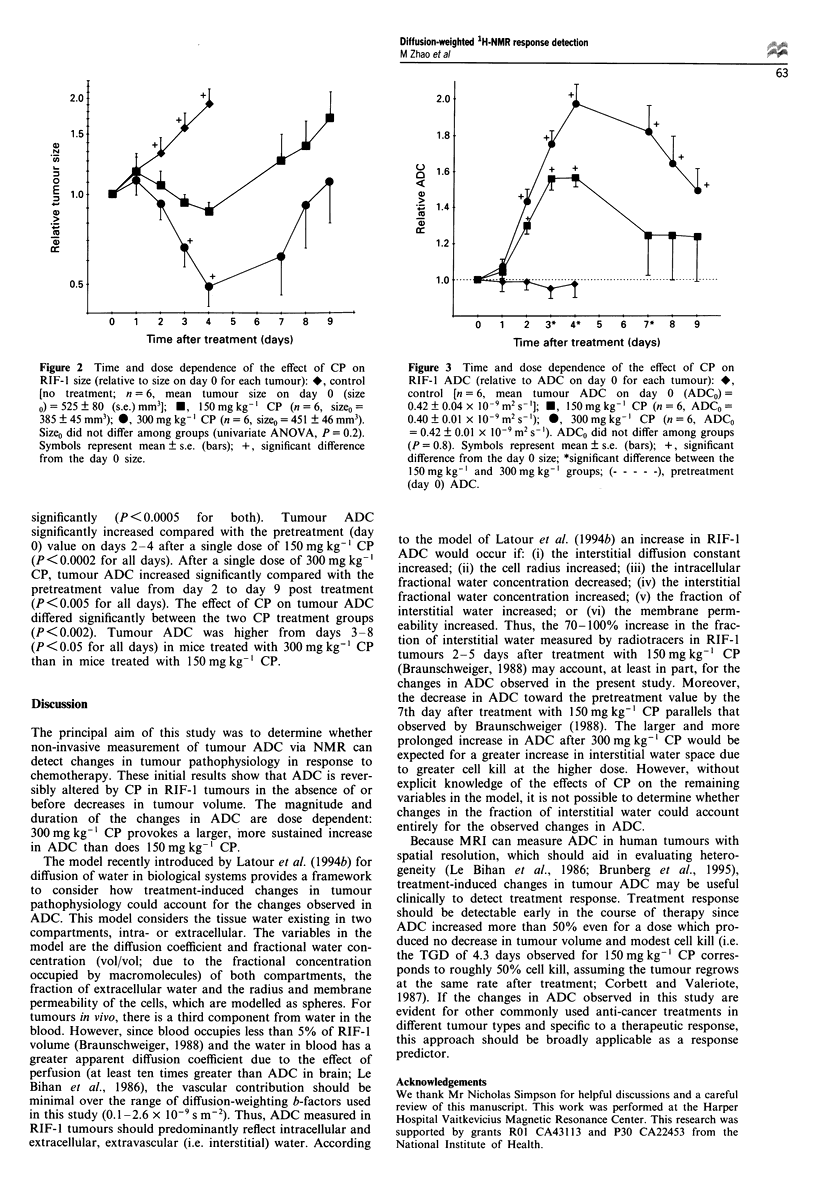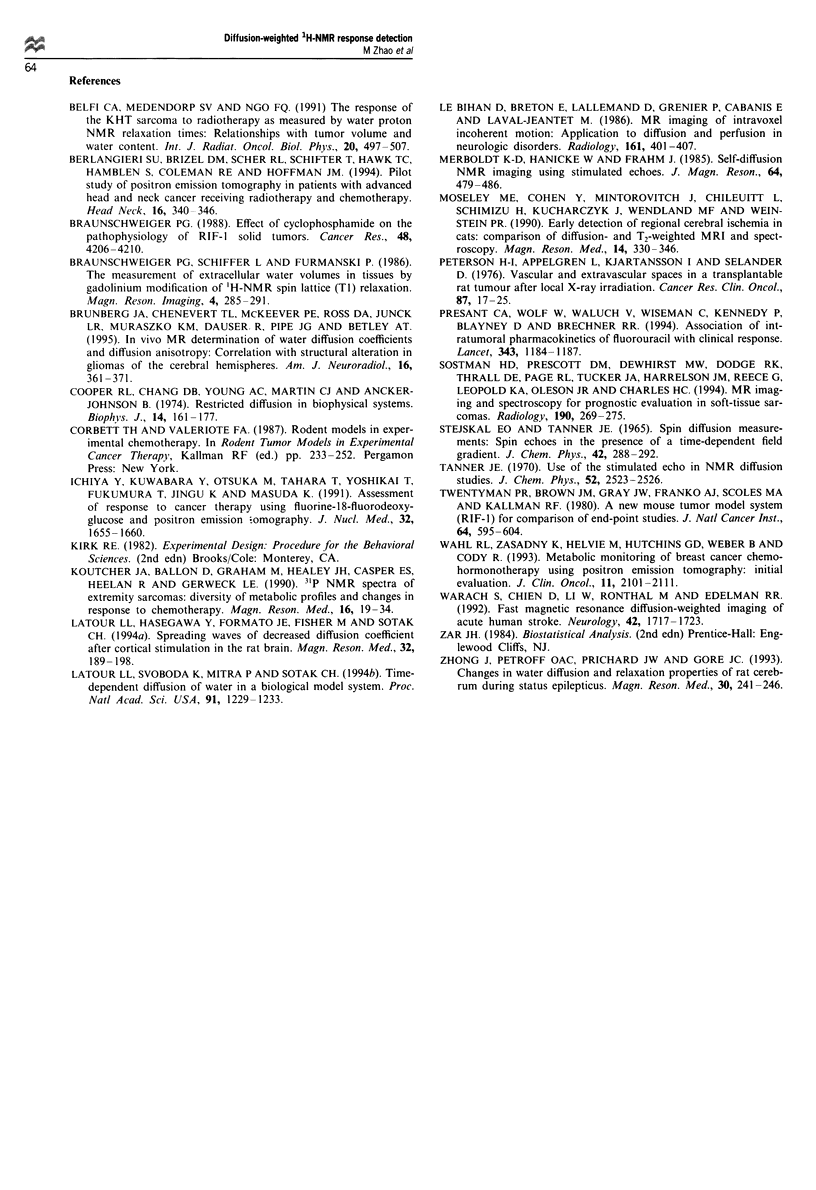# Early detection of treatment response by diffusion-weighted 1H-NMR spectroscopy in a murine tumour in vivo.

**DOI:** 10.1038/bjc.1996.11

**Published:** 1996-01

**Authors:** M. Zhao, J. G. Pipe, J. Bonnett, J. L. Evelhoch

**Affiliations:** Department of Internal Medicine, Wayne State University School of Medicine, Detroit, Michigan 48201, USA.

## Abstract

Nuclear magnetic resonance (NMR) non-invasively measures the apparent diffusion coefficient (ADC) of water, which is sensitive to the biophysical characteristics of tissue. Because anti-cancer treatment alters tumour pathophysiology, tumour ADC may be altered by treatment. In order to test this hypothesis, ADC was measured in s.c. implanted murine RIF-1 tumours before and up to 9 days after treatment with cyclophosphamide. A dose-dependent, reversible increase in tumour ADC was observed after cyclophosphamide treatment, which is consistent with an increase in the fraction of interstitial water due to treatment-induced cell death. Because tumour water ADC is increased substantially at a time when there is no change in tumour volume for a dose which produces minimal cell kill, its measurement could provide a novel means for early detection of response to anti-cancer therapy. If the changes in ADC observed in the present study are evident for commonly used anti-cancer therapies in different tumour types and specific to a therapeutic response, the approach could be broadly applicable as a response predictor since magnetic resonance imaging can be used to measure ADC in human tumours.


British Journal of Cancer (1996) 73, 61-64
© 1996 Stockton Press All rights reserved 0007-0920/96 $12.00

### Early detection of treatment response by diffusion-weighted $^{1}$H-NMR spectroscopy in a murine tumour *in vivo*

M Zhao[1], JG Pipe[2], J Bonnett[1] and JL Evelhoch[1,2]

*Departments of [1]Internal Medicine and [2]Radiology, Wayne State University School of Medicine, Detroit, Michigan 48201, USA.*

**Summary** Nuclear magnetic resonance (NMR) non-invasively measures the apparent diffusion coefficient (ADC) of water, which is sensitive to the biophysical characteristics of tissue. Because anti-cancer treatment alters tumour pathophysiology, tumour ADC may be altered by treatment. In order to test this hypothesis, ADC was measured in s.c. implanted murine RIF-1 tumours before and up to 9 days after treatment with cyclophosphamide. A dose-dependent, reversible increase in tumour ADC was observed after cyclophosphamide treatment, which is consistent with an increase in the fraction of interstitial water due to treatment-induced cell death. Because tumour water ADC is increased substantially at a time when there is no change in tumour volume for a dose which produces minimal cell kill, its measurement could provide a novel means for early detection of response to anti-cancer therapy. If the changes in ADC observed in the present study are evident for commonly used anti-cancer therapies in different tumour types and specific to a therapeutic response, this approach could be broadly applicable as a response predictor since magnetic resonance imaging can be used to measure ADC in human tumours.

**Keywords:** therapeutic response; tumour pathophysiology; tumour water; nuclear magnetic resonance; RIF-1

The response of human malignancies to commonly used therapy varies markedly even for patients with tumours of the same tissue of origin, stage and classification. Clinical detection of therapeutic efficacy (generally a 50% decrease in tumour volume assessed radiographically) is seldom possible early in the course of treatment when the information could be used to tailor therapy for individual patients. Thus, the need for early predictors of tumour responsiveness to commonly used therapeutic modalities has long been recognised. The ability to predict therapeutic response in individual patients after an initial (or 'test') dose of a fraction of the maximum tolerated dosage would both allow patients with non-responsive tumours to avoid the side-effects and toxicities of a full course of therapy and aid in the selection of patients for the clinical trials of new anti-tumour therapies.

One approach being evaluated as a response predictor is to assess tumours before and during treatment in an attempt to detect changes in the pathophysiology of tumours responding to the initial therapy. Non-invasive methods, including magnetic resonance spectroscopy (e.g. Koutcher *et al.*, 1990; Presant *et al.*, 1994; Sostman *et al.*, 1994) and positron emission tomography (e.g. Ichiya *et al.*, 1991; Wahl *et al.*, 1993; Berlangieri *et al.*, 1994), are being evaluated for their ability to provide such information. While the initial results using these approaches are encouraging, their ultimate utility remains to be established. Potential weaknesses of these approaches are their limited sensitivity to therapeutic response and coarse spatial resolution. Ideally, one would like to be able to detect the changes associated with a small therapeutic effect at the highest possible spatial resolution. This would both permit intra-tumour heterogeneity to be assessed and allow the smallest possible 'test' dose to be used to identify patients with non-responsive tumours. Although magnetic resonance imaging (MRI) has the spatial resolution needed to assess macroscopic heterogeneity, treatment-induced changes in relaxation times have been reported only for *ex vivo* measurements (Braunschweiger *et al.*, 1986; Belfi *et al.*, 1991) and they may not provide sufficient sensitivity to serve as response predictors. However, recent reports of the ability of MRI to detect changes in brain water diffusion associated with pathophysiological changes occurring as a result of cerebral ischaemia (Mosely *et al.*, 1990; Warach *et al.*, 1992), *status epilepticus* (Zhong *et al.*, 1993) or spreading cortical depression (Latour *et al.*, 1994*a*) indicate that diffusion measurements may provide greater sensitivity to changes in tumour pathophysiology in response to treatment.

Water diffusion measured by pulsed-field gradient nuclear magnetic resonance (NMR; Stejskal and Tanner, 1965) is influenced by the restriction of diffusion due to the limited permeability of cell membranes to water (Cooper *et al.*, 1974). Hence, the water diffusion coefficient measured by NMR is referred to as the *apparent* diffusion coefficient (ADC) and it is sensitive to the biophysical characteristics of tissue, including the fraction of water in the extracellular space (Latour *et al.*, 1994*b*). Because both radiation (Peterson *et al.*, 1976) and chemotherapy (Braunschweiger, 1988) substantially increase the fraction of extracellular water, tumour water ADC may be altered by treatment. If therapy causes a change in tumour water ADC, it could provide a clinically applicable therapeutic response predictor because MRI of water diffusion can be applied to human tumours (Le Bihan *et al.*, 1986; Brunberg *et al.*, 1995). Consequently, in an effort to determine if treatment-induced changes in tumour pathophysiology alter tumour water ADC before a significant decrease in tumour size, ADC was measured in s.c. implanted murine radiation-induced fibrosarcoma 1 (RIF-1) tumours before and up to 9 days after treatment with cyclophosphamide (CP; 150 mg $kg^{-1}$ or 300 mg $kg^{-1}$).

### Materials and methods

### *Murine tumour model*

The RIF-1 originally obtained from RF Kallman (Stanford University) was maintained by serial *in vivo–in vitro* passage as described by Twentyman *et al.* (1980). Tumours were implanted in female $C_3H$/HeJ mice (Harlan Sprague-Dawley, Indianapolis, IN, USA) by s.c. injecting $10^5$ cultured cells in 0.1 ml of medium into the shaved back of the animal about 2 cm from the tail base. Tumour sizes were estimated by calculating the volume of an ellipsoid $V = (\pi/6)abc$ where $a$, $b$, and $c$ are three perpendicular axes determined from caliper measurements. When tumours reached 200–600 $mm^3$, mice were distributed into one of three treatment groups: control (no treatment); 150 mg $kg^{-1}$ CP; or 300 mg $kg^{-1}$ CP. After NMR measurement of tumour ADC (day 0), mice in the CP

Correspondence: M Zhao, MR Center/Concourse, Harper Hospital, 3990 John R., Detroit, MI 48201, USA
Received 5 April 1995; revised 25 July 1995; accepted 15 August 1995

treatment groups received an i.p. injection of freshly prepared CP in 0.2 ml of sterile water. Tumour response to treatment was estimated by the tumour growth delay (TGD, i.e. median time for treated tumours to double in size minus the median time for control tumours to double in size; Corbett and Valeriote, 1987).

### *NMR measurements*

NMR spectra were acquired on a Bruker Biospec II spectrometer equipped with a 4.7-T horizontal-bore magnet and actively shielded gradients. During NMR observation, the mouse was anaesthetised (1% v/v halothane) and immobilised on a bed that was heated by circulating temperature-controlled water. A thermocouple was inserted rectally to monitor mouse core temperature (36.5 ± 0.5°C). The tumour was isolated from the mouse body using a copper shield with a variable diameter hole to minimise signal contamination from normal tissue. Diffusion-weighted $^1$H-NMR spectra were acquired using a pulsed-field gradient, stimulated echo (PFGSTE) pulse sequence (Tanner, 1970). Four single-acquisition echoes were acquired for each $b$-factor using a repetition time of 10 s to avoid saturation effects. The diffusion-weighted $b$-factor ($b = \gamma^2G^2\delta^2$ $(\Delta - \delta/3)$) was varied by changing the diffusion gradient strength ($G$) from 10 to 30 mT m$^{-1}$, while the duration of the diffusion gradient ($\delta$) and the gradient separation time ($\Delta$) were held constant at 10 ms and 200 ms respectively. The anisotropy of tumour ADC was tested by repeating the measurements in six untreated tumours using the $x$, $y$, or $z$ gradient as the diffusion gradient.

### *Data analysis*

For a given $b$-factor, each of the single acquisition echoes were baseline-corrected, zero-filled, Fourier-transformed, and magnitude spectra were calculated. The water PFGSTE amplitudes were measured as peak heights from the four magnitude spectra and the amplitudes were averaged to avoid phase-related motion artifacts. Model fitting and statistical analyses were performed using CSS:STATISTICA/w (StatSoft, Tulsa, OK, USA). Tumour ADC was estimated by non-linear least squares fitting (Quasi-Newton Algorithm) of the PFGSTE amplitude as a function of the diffusion-weighting $b$-factor to the Stejskal–Tanner formula (Stejskal and Tanner, 1965), which relates the echo amplitude for a given $b$-factor ($A_b$) to ADC; $A_b = A_0 \times \exp(-b \times \text{ADC})$.

**Figure 1** Representative semi-log plots of RIF-1 tumour water PFGSTE amplitudes (arbitrary units) as a function of the diffusion-weighting $b$-factor. Symbols are measured amplitudes for: ◆, control tumour, ADC = 0.41 ± 0.01 × 10$^{-9}$ m$^2$ s$^{-1}$ (s.e. estimated from fit); ■, tumour 4 days after 150 mg kg$^{-1}$ CP, ADC = 0.61 ± 0.02 × 10$^{-9}$ m$^2$ s$^{-1}$; and ●, tumour 4 days after 300 mg kg$^{-1}$ CP, ADC = 0.83 ± 0.02 × 10$^{-9}$ m$^2$ s$^{-1}$. The lines correspond to non-linear least squares fits of these data to the Stejskal–Tanner formula.

### *Statistics*

Two dependent variables measured across time were analysed: tumour size and tumour ADC. Both variables were measured five times in mice in the no treatment group (days 0, 1, 2, 3 and 4) and eight times in mice in the CP treatment groups (before treatment and days 1–4 and 7–9 after treatment). For both variables, the following analyses were performed. First, a repeated measures two-factor (time, treatment group) analysis of variance (ANOVA) using Geisser–Greenhouse-corrected $P$-values was performed (Kirk, 1982). When a statistically significant interaction was detected between treatment group and time, a repeated measures ANOVA was performed to test for change over time in each treatment group. If a significant change over time was detected, a Tukey test (Zar, 1984) was performed to determine at which time points the value differed from the pretreatment (day 0) value. A repeated measures two-factor (time, treatment group) ANOVA using Geisser–Greenhouse-corrected $P$-values was also performed for the data from the two CP treatment groups. When a statistically significant interaction was detected between the CP dose and time, a Tukey test was performed to determine at which time points the value differed between the CP doses.

### Results

The ADC of tap water in a flask at 20 ± 1°C was measured by the PFGSTE pulse sequence to be 2.15 ± 0.02 (10$^{-9}$ m$^2$ s$^{-1}$), which is consistent with the literature value (Merboldt *et al*., 1985). Figure 1 shows representative PFGSTE data from which tumour water ADC was determined for a control (no treatment) RIF-1 tumour and for RIF-1 tumours 4 days after a single dose of either 150 mg kg$^{-1}$ or 300 mg kg$^{-1}$ CP. The tumour water PFGSTE amplitudes as a function of the diffusion-weighting $b$-factor were fitted to the Stejskal–Tanner formula (Stejskal and Tanner, 1965) to derive tumour ADC: these ranged from 0.33 × 10$^{-9}$ m$^2$ s$^{-1}$ to 0.96 × 10$^{-9}$ m$^2$ s$^{-1}$ in all measurements. The maximum difference between tumour ADC measured with the diffusion gradient oriented along the $x$, $y$ or $z$-axis of the magnetic field was ± 0.01 × 10$^{-9}$ m$^2$ s$^{-1}$ in six tumours. Consequently, tumour ADC is isotropic in RIF-1 tumours.

The time and dose dependence of the size of RIF-1 tumours treated with CP is presented in Figure 2. The pattern of changes over time in tumour size differed among the treatment groups (control, 150 mg kg$^{-1}$ CP and 300 mg kg$^{-1}$ CP) over the first four days of observation (two-factor repeated measures ANOVA; $P < 0.00001$ for the interaction between time and dose). In control tumours the size increased significantly over 4 days of observation (repeated measures ANOVA; $P = 0.0004$). The tumour size increased significantly compared with the day 0 size on days 2–4 (Tukey test; $P < 0.001$ for all days). Although tumour size did not change significantly over time after treatment with 150 mg kg$^{-1}$ CP ($P > 0.1$), treatment with 300 mg kg$^{-1}$ did significantly change tumour size ($P < 0.00005$). Tumour size decreased significantly compared with the pretreatment value on days 3 and 4 after a single dose of 300 mg kg$^{-1}$ CP, ($P < 0.0002$ for both days) and returned to a size not significantly different from the pretreatment size by day 7. The effect of CP on tumour size did not differ significantly between the two CP treatment groups ($P > 0.1$).

The time and dose dependence of the ADC in RIF-1 tumours treated with CP is presented in Figure 3. The pattern of changes over time in tumour ADC differed among the treatment groups ($P < 0.00001$ for the interaction between time and dose) over the first 4 days of observation. In control tumours, ADC did not change ($P > 0.6$) over 4 days of observation. However, over time after treatment with either 150 mg kg$^{-1}$ CP or 300 mg kg$^{-1}$ CP, tumour ADC changed

**Figure 2** Time and dose dependence of the effect of CP on RIF-1 size (relative to size on day 0 for each tumour): ◆, control [no treatment; $n = 6$, mean tumour size on day 0 (size $_0$) = 525 ± 80 (s.e.) mm$^3$]; ■, 150 mg kg$^{-1}$ CP ($n = 6$, size$_0$ = 385 ± 45 mm$^3$); ●, 300 mg kg$^{-1}$ CP ($n = 6$, size$_0$ = 451 ± 46 mm$^3$). Size$_0$ did not differ among groups (univariate ANOVA, $P = 0.2$). Symbols represent mean ± s.e. (bars); +, significant difference from the day 0 size.

**Figure 3** Time and dose dependence of the effect of CP on RIF-1 ADC (relative to ADC on day 0 for each tumour): ◆, control [$n = 6$, mean tumour ADC on day 0 (ADC$_0$) = 0.42 ± 0.04 × 10$^{-9}$ m$^2$ s$^{-1}$]; ■, 150 mg kg$^{-1}$ CP ($n = 6$, ADC$_0$ = 0.40 ± 0.01 × 10$^{-9}$ m$^2$ s$^{-1}$); ●, 300 mg kg$^{-1}$ CP ($n = 6$, ADC$_0$ = 0.42 ± 0.01 × 10$^{-9}$ m$^2$ s$^{-1}$). ADC$_0$ did not differ among groups ($P = 0.8$). Symbols represent mean ± s.e. (bars); +, significant difference from the day 0 size; *significant difference between the 150 mg kg$^{-1}$ and 300 mg kg$^{-1}$ groups; (- - - - -), pretreatment (day 0) ADC.

significantly ($P < 0.0005$ for both). Tumour ADC significantly increased compared with the pretreatment (day 0) value on days 2–4 after a single dose of 150 mg kg$^{-1}$ CP ($P < 0.0002$ for all days). After a single dose of 300 mg kg$^{-1}$ CP, tumour ADC increased significantly compared with the pretreatment value from day 2 to day 9 post treatment ($P < 0.005$ for all days). The effect of CP on tumour ADC differed significantly between the two CP treatment groups ($P < 0.002$). Tumour ADC was higher from days 3–8 ($P < 0.05$ for all days) in mice treated with 300 mg kg$^{-1}$ CP than in mice treated with 150 mg kg$^{-1}$ CP.

### Discussion

The principal aim of this study was to determine whether non-invasive measurement of tumour ADC via NMR can detect changes in tumour pathophysiology in response to chemotherapy. These initial results show that ADC is reversibly altered by CP in RIF-1 tumours in the absence of or before decreases in tumour volume. The magnitude and duration of the changes in ADC are dose dependent: 300 mg kg$^{-1}$ CP provokes a larger, more sustained increase in ADC than does 150 mg kg$^{-1}$ CP.

The model recently introduced by Latour *et al.* (1994*b*) for diffusion of water in biological systems provides a framework to consider how treatment-induced changes in tumour pathophysiology could account for the changes observed in ADC. This model considers the tissue water existing in two compartments, intra- or extracellular. The variables in the model are the diffusion coefficient and fractional water concentration (vol/vol; due to the fractional concentration occupied by macromolecules) of both compartments, the fraction of extracellular water and the radius and membrane permeability of the cells, which are modelled as spheres. For tumours *in vivo*, there is a third component from water in the blood. However, since blood occupies less than 5% of RIF-1 volume (Braunschweiger, 1988) and the water in blood has a greater apparent diffusion coefficient due to the effect of perfusion (at least ten times greater than ADC in brain; Le Bihan *et al.*, 1986), the vascular contribution should be minimal over the range of diffusion-weighting *b*-factors used in this study (0.1–2.6 × 10$^{-9}$ s m$^{-2}$). Thus, ADC measured in RIF-1 tumours should predominantly reflect intracellular and extracellular, extravascular (i.e. interstitial) water. According to the model of Latour *et al.* (1994*b*) an increase in RIF-1 ADC would occur if: (i) the interstitial diffusion constant increased; (ii) the cell radius increased; (iii) the intracellular fractional water concentration decreased; (iv) the interstitial fractional water concentration increased; (v) the fraction of interstitial water increased; or (vi) the membrane permeability increased. Thus, the 70–100% increase in the fraction of interstitial water measured by radiotracers in RIF-1 tumours 2–5 days after treatment with 150 mg kg$^{-1}$ CP (Braunschweiger, 1988) may account, at least in part, for the changes in ADC observed in the present study. Moreover, the decrease in ADC toward the pretreatment value by the 7th day after treatment with 150 mg kg$^{-1}$ CP parallels that observed by Braunschweiger (1988). The larger and more prolonged increase in ADC after 300 mg kg$^{-1}$ CP would be expected for a greater increase in interstitial water space due to greater cell kill at the higher dose. However, without explicit knowledge of the effects of CP on the remaining variables in the model, it is not possible to determine whether changes in the fraction of interstitial water could account entirely for the observed changes in ADC.

Because MRI can measure ADC in human tumours with spatial resolution, which should aid in evaluating heterogeneity (Le Bihan *et al.*, 1986; Brunberg *et al.*, 1995), treatment-induced changes in tumour ADC may be useful clinically to detect treatment response. Treatment response should be detectable early in the course of therapy since ADC increased more than 50% even for a dose which produced no decrease in tumour volume and modest cell kill (i.e. the TGD of 4.3 days observed for 150 mg kg$^{-1}$ CP corresponds to roughly 50% cell kill, assuming the tumour regrows at the same rate after treatment; Corbett and Valeriote, 1987). If the changes in ADC observed in this study are evident for other commonly used anti-cancer treatments in different tumour types and specific to a therapeutic response, this approach should be broadly applicable as a response predictor.

**Acknowledgements**
We thank Mr Nicholas Simpson for helpful discussions and a careful review of this manuscript. This work was performed at the Harper Hospital Vaitkevicius Magnetic Resonance Center. This research was supported by grants R01 CA43113 and P30 CA22453 from the National Institute of Health.

### References

BELFI CA, MEDENDORP SV AND NGO FQ. (1991) The response of the KHT sarcoma to radiotherapy as measured by water proton NMR relaxation times: Relationships with tumor volume and water content. *Int. J. Radiat. Oncol. Biol. Phys.*, **20,** 497–507.

BERLANGIERI SU, BRIZEL DM, SCHER RL, SCHIFTER T, HAWK TC, HAMBLEN S, COLEMAN RE AND HOFFMAN JM. (1994). Pilot study of positron emission tomography in patients with advanced head and neck cancer receiving radiotherapy and chemotherapy. *Head Neck*, **16,** 340–346.

BRAUNSCHWEIGER PG. (1988). Effect of cyclophosphamide on the pathophysiology of RIF-1 solid tumors. *Cancer Res.*, **48,** 4206–4210.

BRAUNSCHWEIGER PG, SCHIFFER L AND FURMANSKI P. (1986). The measurement of extracellular water volumes in tissues by gadolinium modification of $^1$H-NMR spin lattice (T1) relaxation. *Magn. Reson. Imaging*, **4,** 285–291.

BRUNBERG JA, CHENEVERT TL, MCKEEVER PE, ROSS DA, JUNCK LR, MURASZKO KM, DAUSER R, PIPE JG AND BETLEY AT. (1995). In vivo MR determination of water diffusion coefficients and diffusion anisotropy: Correlation with structural alteration in gliomas of the cerebral hemispheres. *Am. J. Neuroradiol.*, **16,** 361–371.

COOPER RL, CHANG DB, YOUNG AC, MARTIN CJ AND ANCKER-JOHNSON B. (1974). Restricted diffusion in biophysical systems. *Biophys. J.*, **14,** 161–177.

CORBETT TH AND VALERIOTE FA. (1987). Rodent models in experimental chemotherapy. In *Rodent Tumor Models in Experimental Cancer Therapy*, Kallman RF (ed.) pp. 233–252. Pergamon Press: New York.

ICHIYA Y, KUWABARA Y, OTSUKA M, TAHARA T, YOSHIKAI T, FUKUMURA T, JINGU K AND MASUDA K. (1991). Assessment of response to cancer therapy using fluorine-18-fluorodeoxyglucose and positron emission tomography. *J. Nucl. Med.*, **32,** 1655–1660.

KIRK RE. (1982). *Experimental Design: Procedure for the Behavioral Sciences.* (2nd edn) Brooks/Cole: Monterey, CA.

KOUTCHER JA, BALLON D, GRAHAM M, HEALEY JH, CASPER ES, HEELAN R AND GERWECK LE. (1990). $^{31}$P NMR spectra of extremity sarcomas: diversity of metabolic profiles and changes in response to chemotherapy. *Magn. Reson. Med.*, **16,** 19–34.

LATOUR LL, HASEGAWA Y, FORMATO JE, FISHER M AND SOTAK CH. (1994*a*). Spreading waves of decreased diffusion coefficient after cortical stimulation in the rat brain. *Magn. Reson. Med.*, **32,** 189–198.

LATOUR LL, SVOBODA K, MITRA P AND SOTAK CH. (1994*b*). Time-dependent diffusion of water in a biological model system. *Proc. Natl Acad. Sci. USA*, **91,** 1229–1233.

LE BIHAN D, BRETON E, LALLEMAND D, GRENIER P, CABANIS E AND LAVAL-JEANTET M. (1986). MR imaging of intravoxel incoherent motion: Application to diffusion and perfusion in neurologic disorders. *Radiology*, **161,** 401–407.

MERBOLDT K-D, HANICKE W AND FRAHM J. (1985). Self-diffusion NMR imaging using stimulated echoes. *J. Magn. Reson.*, **64,** 479–486.

MOSELEY ME, COHEN Y, MINTOROVITCH J, CHILEUITT L, SCHIMIZU H, KUCHARCZYK J, WENDLAND MF AND WEINSTEIN PR. (1990). Early detection of regional cerebral ischemia in cats: comparison of diffusion- and $T_2$-weighted MRI and spectroscopy. *Magn. Reson. Med.*, **14,** 330–346.

PETERSON H-I, APPELGREN L, KJARTANSSON I AND SELANDER D. (1976). Vascular and extravascular spaces in a transplantable rat tumour after local X-ray irradiation. *Cancer Res. Clin. Oncol.*, **87,** 17–25.

PRESANT CA, WOLF W, WALUCH V, WISEMAN C, KENNEDY P, BLAYNEY D AND BRECHNER RR. (1994). Association of intratumoral pharmacokinetics of fluorouracil with clinical response. *Lancet*, **343,** 1184–1187.

SOSTMAN HD, PRESCOTT DM, DEWHIRST MW, DODGE RK, THRALL DE, PAGE RL, TUCKER JA, HARRELSON JM, REECE G, LEOPOLD KA, OLESON JR AND CHARLES HC. (1994). MR imaging and spectroscopy for prognostic evaluation in soft-tissue sarcomas. *Radiology*, **190,** 269–275.

STEJSKAL EO AND TANNER JE. (1965). Spin diffusion measurements: Spin echoes in the presence of a time-dependent field gradient. *J. Chem. Phys.*, **42,** 288–292.

TANNER JE. (1970). Use of the stimulated echo in NMR diffusion studies. *J. Chem. Phys.*, **52,** 2523–2526.

TWENTYMAN PR, BROWN JM, GRAY JW, FRANKO AJ, SCOLES MA AND KALLMAN RF. (1980). A new mouse tumor model system (RIF-1) for comparison of end-point studies. *J. Natl Cancer Inst.*, **64,** 595–604.

WAHL RL, ZASADNY K, HELVIE M, HUTCHINS GD, WEBER B AND CODY R. (1993). Metabolic monitoring of breast cancer chemohormonotherapy using positron emission tomography: initial evaluation. *J. Clin. Oncol.*, **11,** 2101–2111.

WARACH S, CHIEN D, LI W, RONTHAL M AND EDELMAN RR. (1992). Fast magnetic resonance diffusion-weighted imaging of acute human stroke. *Neurology*, **42,** 1717–1723.

ZAR JH. (1984). *Biostatistical Analysis.* (2nd edn) Prentice-Hall: Englewood Cliffs, NJ.

ZHONG J, PETROFF OAC, PRICHARD JW AND GORE JC. (1993). Changes in water diffusion and relaxation properties of rat cerebrum during status epilepticus. *Magn. Reson. Med.*, **30,** 241–246.